# P-1613. Blood Culture Contamination Mitigation: Sustained Stewardship Successes Seen Systemwide

**DOI:** 10.1093/ofid/ofae631.1780

**Published:** 2025-01-29

**Authors:** Mark D Povroznik

**Affiliations:** United Hospital Center, Bridgeport, West Virginia

## Abstract

**Background:**

Contamination prevention, when sampling blood for culture, relies on an operator impeccably adhering to skin preparation routines and attentively avoiding cross-contamination opportunities until laboratory personnel attain specimen possession. Operator-reliant diagnostic integrity was challenged throughout the COVID-19 pandemic, which placed unexpected environmental pressures on personnel to the documented detriment of antimicrobial stewardship endeavors and patient outcomes. Clinician interest in shifting some operator burdens to assistive technology subsequently flared; in accordance with literature reports of successful blood culture contamination mitigation when initial-specimen diversion devices were co-opted into protocol, the National Quality Forum and the Centers for Disease Control and Prevention backed a Clinical and Laboratory Standards Institute revision to blood culture sample collection guidelines, recommending their use. This tech-centric tenet was hypothesized to be necessary to sustaining the successful mitigation of blood culture contamination through the stressor-heavy situations wherein education-focused endeavors alone were struggling to substantiate significance.

Antimicrobial Stewardship Intervention Outcome

**Figure:**
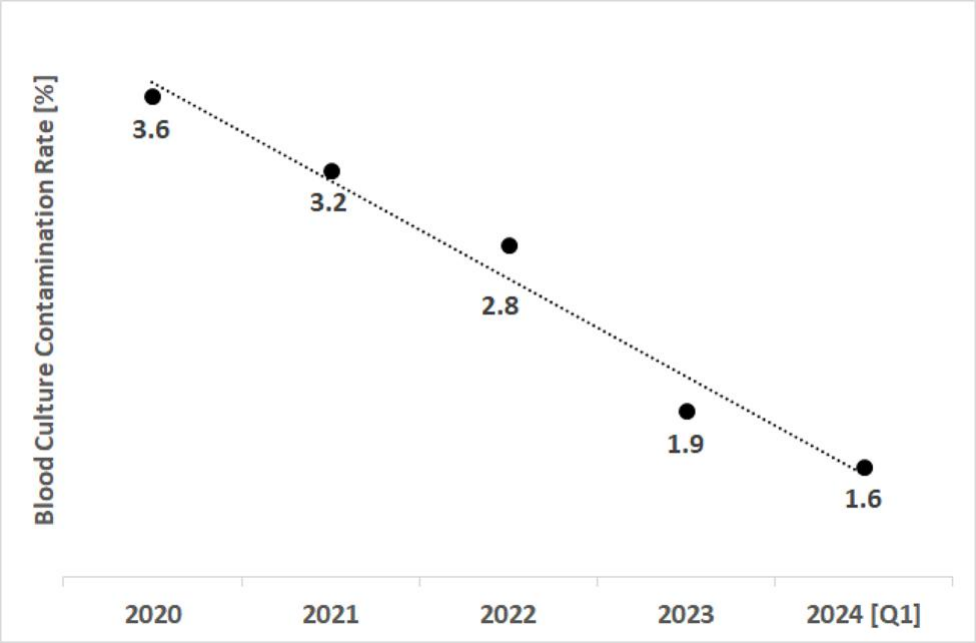

Data reflects approximately 36,000 blood cultures drawn each quarter.

**Methods:**

Intervention began in 2020, prior to which a 3.6% blood culture contamination rate was observed systemwide. Seventeen facilities among nineteen that share a data system co-opted initial-specimen diversion device technology alongside value analysis and cultural curation in a trifurcate strategy to reduce contamination events.

**Results:**

Consistent, real-time communication with caretakers on outcomes enabled year-over-year interventive technology utilization increases [currently approximately 85% systemwide]. The systemwide blood culture contamination rate for the first quarter of 2024 was 1.64% [586 contamination events among 35,750 total blood cultures], down from 1.9% across 2023, 2.8% across 2022, and 3.2% across 2021.

**Conclusion:**

Tech-facilitated closure of contaminant pathways, when paired with communication channels connecting individual contributions to stewardship successes, sustained the year-over-year systemwide reduction in blood culture contamination events observed herein.

**Disclosures:**

**All Authors**: No reported disclosures

